# Supplementary material for: Identifying images in the biology literature that are problematic for people with a color-vision deficiency
Source: eLife. 2024 Sep 11;13:RP95524. doi: 10.7554/eLife.95524 (PMC11390107; doi:10.7554/eLife.95524)
Supplement: Supplementary file 1. — (A) Predictive performance for metrics that characterize potentially problematic aspects of images. We calculated five metrics, as well as a rank-based, combined score and assessed their ability to categorize images as “Definitely okay” or “Definitely problematic”. (B) Predictive performance for classification algorithms that used the image metrics as inputs. We used classification algorithms to categorize images as “Definitely okay” or “Definitely problematic”. These results indicate the algorithms’ performance after cross validation on the training set. (C) Predictive performance for Convolutional Neural Network models that used the images as inputs. We tested 23 model configurations via cross validation on the training set, evaluating each model’s ability to categorize images as “Definitely okay” or “Definitely problematic”. [file elife-95524-supp1.docx]

**Supplementary File 1A**

| **Metric** | **AUROC** | **AUPRC** |
| --- | --- | --- |
| Mean, pixel-wise color distance between the original and simulated image | 0.44 | 0.12 |
| Color-distance ratio between the original and simulated images for the color pair with the largest distance in the original image | 0.63 | 0.19 |
| Number of color pairs that exhibited a high color-distance ratio between the original and simulated images | 0.75 | 0.34 |
| Proportion of pixels in the original image that used a color from one of the high-ratio color pairs | 0.73 | 0.28 |
| Mean Euclidean distance between pixels for high-ratio color pairs | 0.67 | 0.24 |
| Rank-based score that combines the metrics | 0.71 | 0.26 |

**Supplementary File 1B**

| **Algorithm** | **AUROC** | **AUPRC** |
| --- | --- | --- |
| Logistic Regression | 0.82 | 0.43 |
| Nearest Neighbors | 0.72 | 0.32 |
| Random Forests | 0.80 | 0.42 |

**Supplementary File 1C**

| **Combination** | **Class weighting** | **Early stopping** | **Random rotation** | **Dropout** | **Transfer learning** | **Fine tuning** | **AUROC** | **AUPRC** |
| --- | --- | --- | --- | --- | --- | --- | --- | --- |
| 0 | No | No | 0.0 | 0.0 | None | No | 0.77 | 0.48 |
| 1 | Yes | No | 0.0 | 0.0 | None | No | 0.83 | 0.51 |
| 2 | No | Yes | 0.0 | 0.0 | None | No | 0.88 | 0.58 |
| 3 | No | No | 0.2 | 0.0 | None | No | 0.91 | 0.68 |
| 4 | No | No | 0.3 | 0.0 | None | No | 0.91 | 0.68 |
| 5 | No | No | 0.0 | 0.2 | None | No | 0.78 | 0.51 |
| 6 | No | No | 0.0 | 0.5 | None | No | 0.80 | 0.45 |
| 7 | No | No | 0.0 | 0.0 | MobileNetV2 | No | 0.85 | 0.55 |
| 8 | No | No | 0.0 | 0.0 | ResNet50 | No | 0.87 | 0.62 |
| 9 | No | No | 0.0 | 0.0 | MobileNetV2 | Yes | 0.85 | 0.61 |
| 10 | No | No | 0.0 | 0.0 | ResNet50 | Yes | 0.85 | 0.62 |
| 11 | Yes | Yes | 0.2 | 0.0 | ResNet50 | No | 0.89 | 0.62 |
| 12 | Yes | Yes | 0.2 | 0.2 | ResNet50 | No | 0.88 | 0.60 |
| 13 | Yes | Yes | 0.2 | 0.5 | ResNet50 | No | 0.88 | 0.58 |
| 14 | Yes | Yes | 0.2 | 0.0 | ResNet50 | Yes | 0.92 | 0.74 |
| 15 | Yes | Yes | 0.2 | 0.2 | ResNet50 | Yes | 0.92 | 0.74 |
| 16 | Yes | Yes | 0.2 | 0.5 | ResNet50 | Yes | 0.93 | 0.75 |
| 17 | Yes | Yes | 0.2 | 0.0 | MobileNetV2 | No | 0.86 | 0.52 |
| 18 | Yes | Yes | 0.2 | 0.2 | MobileNetV2 | No | 0.86 | 0.52 |
| 19 | Yes | Yes | 0.2 | 0.5 | MobileNetV2 | No | 0.87 | 0.53 |
| 20 | Yes | Yes | 0.2 | 0.0 | MobileNetV2 | Yes | 0.89 | 0.65 |
| 21 | Yes | Yes | 0.2 | 0.2 | MobileNetV2 | Yes | 0.90 | 0.68 |
| 22 | Yes | Yes | 0.2 | 0.5 | MobileNetV2 | Yes | 0.91 | 0.69 |
